# Report of the Wounded at the Battle of Okee-Chobee, Florida, 25th December, 1839

**Published:** 1845-09-01

**Authors:** R. C. Wood

**Affiliations:** Surgeon U. S. Army


### Report of the Wounded at the Battle of Okee-Chobee, Florida, 25th December, 1839.

*Admitted, 4th January*, 1838, *at General Hospital, Fort Brooke.*

Communicated for the Buffalo Medical Journal, by R. C. Wood, M. D., Surgeon U. S. Army.

[Our thanks are due to our correspondent and esteemed friend for his very interesting and valuable report, which, when the severity of some of the injuries are considered, present a degree of success which is certainly most extraordinary; and, indeed, we are inclined to doubt if a parallel can be found in the annals of military surgery. While our readers will appreciate the motives of delicacy which induce Dr. W. to pass so lightly over the agency of the medical attendance in contributing to the general result, they will doubtless be disposed to regard, certainly not last, if not first in importance, the judicious instructions of the senior medical officer and director, together with the fidelity, and vigilant care of his associates of the medical staff.

EDITOR.]

BUFFALO BARRACKS, N. Y.
*My dear Sir:* August 10, 1845.

In the July number of the New-York Journal of Medicine for the present year, there are a few cases of gun-shot wounds reported (19, of which 7 died,) which injuries resulted from the Kensington riots in Philadelphia. As the wounded on that occasion received the most prompt and able surgical aid, I was induced to refer to some notes of gun-shot wounds in Florida, as illustrative of the effects of climate.

Lake Okee-Chobee is about 100 miles from Tampa Bay. In that vicinity the Seminole Indians collected in large numbers, and surrounded by deep morasses and wide covers of trees, deemed themselves safe, and invited attack. In that position they were attacked by a column, composed of the 1st, 4th, and 6th, U. S. Infantry, and volunteers from Missouri, all under the command of Col. now Brevet Brig. Gen'l T. Taylor, and defeated, escaping by the lake in canoes, and abandoning many of their killed. Of the regulars and volunteers, 25 were killed, and 127 wounded; of the former were Col. Thompson, of 6th Inf'y, Col. Gentry, of Missouri Volunteers, with several gallant officers of the 6th Inf'y. The battle was on the 25th December, 1837; on the 26th the wounded remained on the field, and received such attention and professional aid, as the nature of the service permitted. On the 27th they were placed on litters, and ambulances, and conveyed to Tampa Bay, which place they reached on the 4th January, 1838, and were taken into the General Hospital under my care, with several able assistants. The proper appliances and treatment were directed, the men subjected to the most rigid and abstemious diet (½ rations of bread) with simple and light dressings. In this brief notice it will not be necessary to speak of the treatment in detail. Of 59 cases of which I have preserved notes, selected as the most interesting and important, but one died after being received in Hospital—Abram Shaw, of the 6th Inf'y, with fractured femur, very much reduced by diarrhœa which was prevalent; he died some months after the injury. Those of the men permanently disabled were pensioned, the others returned to duty.

I attribute the successful result of these cases—1st, As the most important, the benign influence of the climate of Florida on gunshot wounds. 2d, The very low diet to which they were subjected, with the stringent police of a Military Hospital, together with the excellent supply of medical stores, &c., for the sick in Florida. 3d, It would not be proper to advert to the members of the medical staff in connexion with the above, all of whom are still in service, except to bear testimony to the professional science, and untiring industry of Surgeon Richard Clark, who died in Florida, and to whom the most important of the cases refered to were intrusted. Many who owe their lives and limbs to his skill, will unite with me in lamenting his early death.

The notes appended were taken on the day the men were admitted, in much haste, and are sent you in their original, crude shape. Should you deem them of any importance, they are at your service.

Very cordially yours,

R. C. WOOD,

Surgeon U. S. Army.

---

### Report of the Wounded.

Edward Willis, 4th Infantry—Gun-shot wound in right arm, with fracture of the humerus just above the condyles.

William Drager, 4th Inft.—Gun-shot wound of fore arm, with compound fracture of the radius.

Thomas Harney, 4th Inft.—Gun-shot wound through the thorax, the ball entering between the 8th and 9th ribs on the right side, and coming out between the cartilages of the 4th and 5th ribs on the left side.

Philip Dunning, 4th Inft.—Gun-shot wound—the ball entering at the chin without fracturing it, but merely detaching some small spiculæ of the bone. The ball still remains lodged in the neck. Extracted 6th of January, 1838.

Edward Eaves, 4th Inft.—Gun-shot wound—the ball entering between the 4th and 5th ribs on the anterior part of the left side of the thorax, and coming out on the posterior side of the thorax between the 5th and 6th ribs, a little anterior to the angle.

William Dogherty, 4th Inft.—Gun-shot wound—the ball entering between the 5th and 6th ribs, fracturing the 5th, on the anterior part of the left side of the thorax, and coming out between the heads of the 10th and 11th ribs on the left side.

Daniel Carr, 4th Inft.—Gun-shot wound—in the anterior part of the right leg, the ball passing through the integuments, without injuring the tibia.

William Averill, 4th Inft.—Gun-shot wound in the left forearm—the ball entering about 2 inches below the elbow joint, and coming out on the anterior part of the arm about 4 inches from the joint.

William Johnson, 4th Inft.—Gun-shot wound—the ball entering the axilla of the left side, passing obliquely forwards and downwards, and coming out about the middle of the arm.

John Falvir, 4th Inft.—Gun-shot wound in the abdomen—the ball passing through the parietes of the right hypocondriac region.

Eri Colburn, 4th Inft.—Gun-shot wounds—1st a ball entering between the metacarpal bones of the left hand. 2d a ball entering the neck between the mastoid process and the angle of lower jaw on the left side; its direction cannot be traced. 3d a ball entering the inner side of the right leg about 2 inches below the knee joint. Also two superficial wounds on the left foot.

John Delany, 6th Inft.—Gun-shot wound in the thigh—the ball entering about 2½ inches above the knee joint and rupturing the tendon of the rectus muscle.

Peter Curtis, 6th Inft.—Gunshot-wound in the left hypocondriac region of the abdomen—its course not ascertained.

John Griffith, 6th Inft.—Gun-shot wounds—a ball entering about the middle of the sternum, passing through the integuments without penetrating the thorax, and lodging in the superior part of the right humerus. 2d also a wound in the left hip; the course of the ball not ascertained.

Abraham Shaw, 6th Inft.—Gun-shot wound in the left thigh, with compound fracture of the femur; also wound in 3d and 4th fingers of left hand with fracture of joint of 3d finger, [only fatal case.]

Henry Hergermine, 6th Inft.—Gun-shot wound—the ball entering between the cartilages of the 5th and 6th ribs and remaining lodged in the thorax.

David Dukes, 6th Inft.—Gun-shot wound in the anterior part of the left thigh about the middle. The course of the ball undiscovered. Wound in the left forearm, the ball entering about 2 inches below the elbow joint, and coming out about the middle of the forearm.

David Callison, 6th Inft.—Gun-shot wounds—extensive laceration of the integuments and muscles under the under lower jaw. Wound in the left forearm, the ball entering the inner side of the arm about 3 inches from the elbow joint, passing between the radius and ulna, and coming out on the posterior part of the arm about 3 inches from the wrist. Also a wound in the right arm, the ball entering about 3 inches from the shoulder joint, passing obliquely downwards and backwards, and was extracted opposite the angles of the 8th or 9th ribs.

Jesse Wilson, 6th Inft.—Gun-shot wound—the ball entering between the clavicle and scapula on the right side and descending into the thorax, where it remains. Is troubled with cough and spits up blood.

William Wilson, 1st Inft.—Gun-shot wound in the left knee, the ball entering on the inside, and coming out on the outside, fracturing the patella.

Bartholemew Driskiel, 6th Inft.—Gun-shot wound, the ball penetrating the back opposite the 5th rib, passing under the scapula, and coming out in the right axilla.

Thomas Jennings, corporal 6th Inft.—Superficial gunshot wound in the right arm—wound in the right side of the thorax—ball entering between the 8th and 9th ribs, about their angles, and penetrating the chest; is troubled with cough, and sometimes spits up blood.

William Philips, 1st Inft.—Gunshot wound on the inside of the right thigh—the course of the ball after entering not ascertained.

John Hannasy, 6th Inft.—Gun-shot wound in the left arm—the ball passing through it about the middle, without fracturing the bone.

James Snow, 6th Inft.—Gun-shot wound in the left arm—the ball passing through it about 3 inches from the shoulder joint, detaching two small spiculæ of the bone. Also a wound in the left wrist, the ball not injuring the joint.

Joshua Runner, 6th Inft.—Gun-shot wound in the right fore-arm, with fracture of the radius.

John Durham, 6th Inft.—Gun-shot wound in right arm—the ball fracturing the radius about the middle of the upper third.

Henry M'Crea, 6th Inft.—Gun-shot wound in the right hand, with fracture of 3d finger—the ball coming out in the palm of the hand.

Bernard Smith, 6th Inft.—Gun-shot wound in left fore-arm—the ball passing through the middle of the lower third, between the radius and ulna, detaching some small spiculæ of the former.

Robert Russel, 6th Inft.—Gun-shot wound in right forearm, the ball passing through between the radius and ulna, without injuring them.

William Cunningham, 6th Inft.—Gun-shot wound in left shoulder—the ball passing through the belly of the deltoid muscle, without injuring the bone.

Michael Kelly, 6th Inft.—Gun-shot wound, the ball penetrating through the integuments over the ridge of the scapula, without fracturing the bone.

David Lamb, 6th Inft.—Gun-shot wound through the left arm—the ball passing through opposite the neck of the humerus, without injury of the bone or large blood vessels.

Martin Van Buren, 6th Inft.—Gun-shot wound through the left forearm at the inside of the elbow, a little below the joint, passing through between the radius and ulna, and coming out about the middle of the forearm, on the opposite side.

Patrick Dunn, 6th Inft.—Gun-shot wound in the left hand, the ball penetrating between the metacarpal bones of the 2d and 3d finger; its course not ascertained.

Washington Howard, 6th Inft.—Gun-shot wound in the neck—the ball passing through opposite the 3d cervical vertebra very near the body of that bone.

Thos. Slater, 6th Inft.—Gunshot wound in the left forearm, the ball passing through about the middle, with fracture of the ulna—a superficial wound in the left hypochondriac region of abdomen. A wound in the back, the ball entering between the spines of the 4th and 5th dorsal vetebræ, passing upwards without injuring the spinal column.

Lewis Lachaner, 6th Inft.—Gun-shot wound, the ball entering about the middle of the sternum, passing upwards and backwards under the integuments, under the clavicle, and coming out near the neck of the scapula.

William H. Beerman, 6th Inft.—Gun-shot wound through the left shoulder, the ball passing through the belly of the deltoid muscle; also a wound through the right forearm, about the middle.

Robert M'Kown, 6th Inft.—Gun-shot wound, the ball passing under the integuments on the back of the right hand, without injuring the bone; a wound in the left hand, with fracture of second phalanx of metacarpal bones.

William Hooper, 6th Inft.—Gun-shot wound in left forearm, about the middle.

John M'Gillam, 6th Inft.—Gun-shot wound in face, the ball entering the mouth and passing through the cheek.

Alex'r Holmes, 6th Inft.—Gun-shot wound—the ball entering opposite and fracturing the angle of the lower maxillary bone, passing downwards and backwards, and coming out of the back of the neck, near the 5th cervical vertebra.

Ira Keefer, 6th Inft—Gun-shot wound in left arm, the ball penetrating on the outside, about the middle of the humerus, its course not ascertained; some small spiculæ of the bone were extracted.

James M'Pherson, 6th Inft.—Gun-shot wound on left side of face, the ball passing through the cheek.

John H. Stanley, 6th Inft.—Gun-shot wound, the ball entering opposite the anterior spine of the ilium, passing upwards under the parietes of the abdomen, and coming out at the upper part of the umbilical region.

James Darmody, 6th Inft.—Gun-shot wound, the ball passing through the belly of the biceps muscle of left arm, and then entering the integuments of the thorax under which it was lodged; the ball was extracted: also a wound on the head, merely through the pericranium.

Thomas Taylor, Mo. Vol.—Gun-shot wound through posterior part of thigh.

Golden Wassun, Mo. Vol—Gun-shot wound through left leg, entering on the outside and passing through.

Parkinson Hawks, Mo. Vol.—Gun-shot wound—ball entering at the superior costa of the scapula, passing downwards, and coming out at the lower and posterior part of the scapula.

William Drurkard, Mo. Vol.—Gun-shot wound—superficial injury of clavicle.

Lorenzo Cottle, Mo. Vol.—Gun-shot wound—ball entering at the sternal extremity of clavicle, and coming out at the anterior process of the scapula.

Harden Wilkerson, Mo. Vol.—Gun-shot wound through the upper part of the left arm.

James Carman, Mo. Vol.—Gun-shot wound—ball entering the upper and inside of thigh.

John Spear, 6th Inft.—Gun-shot wound—ball entering at the 2d rib and coming out at the lower and posterior of the scapula. Attended with difficulty of breathing, cough and spitting of blood.

John Kennedy, 6th Inft. Gun-shot wound through elbow joint of left arm.

James Barnet, 6th Inft.—Gun-shot wound—ball entering the outside of the left arm.

William Moreau, 6th Inft.—Gun-shot wound through right arm.

Powel, 1st Inft.—Gun-shot wound—ball entering at the superciliary ridge of parietal bone, and coming out at mastoid process of the temporal bone.

[☞ An error of date in the caption of the foregoing article escaped notice in correction of the proof. The battle was fought in 1837 not 1839. In the last line of same page for WIDE COVER OF TREES read UNDER COVER OF TREES.]

